# Perceived quality of life and associated lifestyle practices among older people living with HIV in Uganda. A cross-sectional study

**DOI:** 10.1007/s13337-025-00938-6

**Published:** 2025-09-03

**Authors:** Christine Atuhairwe, Cyprian Misinde, Titus Ochieng, Leonard Atuhaire

**Affiliations:** 1 Department of Population Studies, School of Statistics and Planning, College of Business and Management Sciences, Makerere University, Kampala, Uganda; 2 Department of Planning and Applied Statistics, School of Statistics and Planning, College of Business and Management Sciences, Makerere University, Kampala, Uganda; 3 Makerere University, Kampala, Uganda; 4 Cabin Uganda Limited, Kampala, Uganda

**Keywords:** HIV, Older persons, Quality of life, Uganda

## Abstract

Perceived quality of life (QoL) is a critical element for older persons living with HIV-positive diagnosis. The intersection of aging, chronic illness, and the complexities of managing HIV significantly influences their quality of life. This study investigated the relationship between perceived QoL and lifestyle factors among older adults living with HIV in Uganda. This cross-sectional survey was conducted with older adults (above 50 years) living with HIV attending The AIDS Support Organization centres across the major regions in Uganda. Data were cleaned and analyzed using STATA 15, with modified Poisson regression assessing factors associated with perceived quality of life. A total of 440 participants were interviewed. While 52% reported poor perceived quality of life before initiating antiretroviral therapy (ART), this decreased to 35% after ART initiation. This suggests a substantial improvement in the health of older adults living with HIV. Before ART initiation, poor quality of life was significantly associated with being aged 60 years or older (adjusted risk ratio [aRR] 1.17, 95% CI 0.94-1.46), adhering to the Seventh-day Adventist (SDA) faith (aRR 1.89, 95% CI 1.12-3.21), being a casual labourer (aRR 0.62, 95% CI 0.41-0.94), being married (aRR 0.65, 95% CI 0.42-1.01), having one sexual partner (aRR 1.50, 95% CI 1.16-1.93), alcohol consumption (aRR 0.71, 95% CI 0.15-0.85), infrequent fruit consumption (aRR 1.75, 95% CI 0.99-3.09), consuming white meat 1-3 times per week (aRR 1.61, 95% CI 1.23-2.10), engaging in sports 4-7 days per week (aRR 0.25, 95% CI 0.08-0.75), walking 4-7 days per week (aRR 0.79, 95% CI 0.62-1.00), and having diabetes (aRR 1.57, 95% CI 1.13-2.19). After ART initiation, poor quality of life remained significantly associated with being 60 years or older (aRR 1.34, 95% CI 0.98-1.83) and being a salaried employee (aRR 0.38, 95% CI 0.21-0.81). Additionally, frequent white meat consumption (4-7 days per week) (aRR 2.01, 95% CI 1.18-3.42) and moderate sports engagement (1-3 days per week) (aRR 1.94, 95% CI 1.47-2.55) were significantly associated with poorer quality of life after ART initiation. No significant associations were observed with other variables. Perceived quality of life in older persons living with HIV was poorer among those aged over 60 years. The risk of poor QoL was lower among those who eat white meat and participate in sports weekly. Increasing physical activity through regular exercise routines in older adults can help reduce the risk of age-related diseases, improve energy levels, reduce stress, enhance appetite and sleep quality, boost cardiovascular and pulmonary endurance, and enhance overall quality of life.

## Introduction

Perceived quality of life (QoL) reflects an individual’s subjective well-being and capacity to enhance it through lifestyle modifications [8, 13]. These behavioural modifications including healthy eating habits, sexual health, mental and emotional well-being, and reduced tobacco and alcohol use, directly impact QoL [8, 13].

A positive mindset empowers PLHIV to make informed health choices such as adhering to an HIV care plan, maintaining a balanced diet, engaging in exercise, minimizing alcohol consumption and stress, and prioritizing emotional and spiritual well-being, which are crucial for a fulfilling life [1, 9]. This mindset fosters treatment adherence, promotes safe sexual practices, encourages healthy eating habits, and ultimately contributes to a longer and healthier life for PLHIV while reducing the spread of the virus [1]. Identifying negative triggers and actively participating in social support groups are key strategies to improve the overall health and longevity [10, 31]. The projected proportion of older adults living with HIV is likely to exceed 70% by 2030, assessing and supporting their QoL within the context of HIV and related infections becomes key in public health intervention [4, 21, 32]. Despite the benefits of Antiretroviral Therapy (ART), the current QoL across all age groups in Uganda stands at 85%, falling short of the 95% target [4]. Therefore, this study aimed to examine the perceived quality of life of older adults living with HIV who are attending The AIDS Support Organization in Uganda.

## Methods

### Study design

A cross-sectional survey was conducted from February 2023 to December 2024 at 11 TASO Centers of Excellence (COEs) located across major regions of Uganda, including Entebbe, Gulu, Jinja, Masaka, Masindi, Mbale, Mbarara, Mulago, Rukungiri, Soroti, and Tororo.

### Study setting

Founded in 1987 as a non-governmental organization (NGO), TASO was established to address the HIV epidemic in Uganda. Over the years, TASO has evolved from providing social support to offering comprehensive care and treatment services for people living with HIV and AIDS. TASO COEs serve as major providers of ART in Uganda. These services are free and are primarily located within major public health facilities nationwide. Individuals can access these services through walk-in clinics and, based on available resources, periodically receive supplementary support such as seedlings, livestock, and food rations [28].

### Study population, sample size and sampling procedures

This study included only OPLHIV (≥50 years) enrolled in and actively receiving HIV care at the TASO COEs. A sample size of 440 participants was calculated using Yamane’s formula, considering a 5% margin of error and an estimated HIV prevalence of 6.4% in Uganda [5]. The ages of the participants were confirmed using ART clinic files at each centre. To ensure representative sampling, a proportionate allocation method was employed to determine the number of participants to be randomly selected from each of the eleven TASO centres. Trained research assistants then used simple random sampling from the older persons >50 years attending the clinic. Eligibility criteria included being an older adult living with HIV, actively receiving care at a TASO centre, and the ability to communicate in any local language or English. All eligible and willing participants were included in the study.

### Data collection

Data was collected from February 2023 to December 2024 at the 11 TASO COEs. Before data collection, the study was explained to the older adults living with HIV, and their informed consent was obtained. A structured questionnaire was used for data collection. To ensure data quality, the questionnaire was piloted at TASO ART clinics before full-scale data collection. Trained TASO counsellors served as research assistants to assist with data collection.

### Data analysis

Data analysis was done using STATA-15, with the primary outcome of interest being perceived QoL. Among OPLHIV, QoL encompasses optimizing health outcomes and promoting dignified ageing. The assessment of perceived QoL before and after the start on antiretroviral medication included five key indicators from the EuroQol-5D-5L tool: mobility, self-care, ability to perform usual activities, pain/discomfort, and anxiety/depression. Each indicator was measured using a three-point Likert scale: 1-No problems, 2-Some problems, 3-Extreme problems [7, 10, 17]. Subsequently, the overall QoL was categorized into a dichotomous variable: 0-Normal (No problem) and 1-Poor (any problem).

The socio-demographic variables assessed included gender (male, female), age group (50-55, 56-60, and 60+ years), level of education (none, primary, secondary, and tertiary), religious affiliation (Roman Catholic, Protestant, Muslim, and others), marital status (married, divorced, and widowed), number of current sexual partners (none, one, and two or more), employment status (employed, unemployed), and primary livelihood activities (crop cultivation, animal rearing, retail trading).

Lifestyle factors assessed included physical activity (sweeping, gardening, mopping, bicycling, carrying farm produce); fruit consumption (mangoes, oranges, jackfruit, pineapple, apples, guava, yellow bananas; frequency: none, 1-3 days/week, 4-7 days/week); green vegetable consumption (Nakaati, Dodo, Cabbage, Spinach, Sukuma wiki; frequency: none, 1-3 days/week, 4-7 days/week); red meat consumption (1-3 days/week, 4-7 days/week); white meat consumption (chicken, fish, duck, turkey; frequency: none, 1-3 days/week, 4-7 days/week); travel methods involving bicycling or walking (frequency: none, 1-3 days/week, 4-7 days/week); and recreational activities such as sports participation (no, yes), weekly exercise (no, 1-3 days/week, 4-7 days/week), and alcohol consumption (no, yes).

Descriptive statistics, utilizing frequencies, were employed to characterize the study sample and determine the proportion of participants with poor perceived QoL. Modified Poisson regression models were fitted to assess the association between lifestyle factors and perceived QoL. Statistical significance was determined using a p-value threshold of ≤ 0.05 in all tests. Multivariable modified Poisson regression analysis was conducted to identify factors independently associated with poor perceived QoL among OPLHIV.

## Results

Before initiating antiretroviral therapy (ART), 48% of people living with HIV (PLHIV) perceived their quality of life as normal, while 52% did not. After starting ART, 65% of PLHIV perceived their quality of life as normal, compared to 35% who did not. The results suggest that ART is effective in improving the overall well-being and perceived quality of life for many people living with HIV.

Table 1 presents the demographic and lifestyle characteristics of older adults living with HIV. Females constituted 67% of the cohort. The mean age of participants was 58.3 ± 6.8 years, with a median age of 57 years (interquartile range [IQR]: 53–62 years). Forty-four percent of participants were aged 50-55 years. A significant proportion (46%) were widowed, and only 30% reported having one sexual partner. Fifty-two percent of participants had attained a primary level of education, and 40% identified as Roman Catholic. More than half (68%) of the participants were unemployed. Regarding their livelihoods, 58% engaged in subsistence-level food crop cultivation. Bivariate analysis using the chi-square test revealed significant associations between perceived quality of life and age (*p* = 0.06), education status (*p* = 0.01), marital status (*p* = 0.01), religious affiliation (*p* = 0.05), and livelihood activities (*p* < 0.01) among older adults living with HIV. All factors, except age, had a p-value less than 0.05 indicating statistically significant relationships as bolded in Table 1.

**Table 1:** Quality of life and associated demographic factors of older persons living with HIV

		Perceived quality of living in older persons with HIV					
		Before	After				
Category	All	Normal	Poor	p-value	Normal	Poor	p-value
Gender	N (%)	n(%)	n(%)		n(%)	n(%)	
Male	146 (33.1)	74 (35.4)	72 (31.2)	0.346	99 (34.7)	47 (30.3)	0.348
Female	294 (66.9)	135 (64.6)	159 (68.8)		186 (65.3)	108 (69.7)	
Age in years							
50-55 Years	192 (43.6)	103 (49.3)	89 (38.5)	**0.067**	127 (44.6)	65 (41.9)	0.429
56-60 Years	117 (26.6)	52 (24.9)	65 (28.1)		79 (27.7)	38 (24.5)	
>60 Years	131 (29.8)	54 (25.8)	77 (33.3)		79 (27.7)	52 (33.5)	
Level of education							
None	91 (20.7)	32 (15.3)	59 (25.5)	**0.013**	58 (20.4)	33 (21.3)	0.276
Completed primary level	228 (51.8)	107 (51.2)	121 (52.4)		140 (49.1)	88 (56.8)	
Completed secondary level	89 (20.2)	52 (24.9)	37 (16.0)		64 (22.5)	25 (16.1)	
Completed tertiary level	32 (7.3)	18 (8.6)	14 (6.1)		23 (8.1)	9 (5.8)	
Religious affiliation							
Roman catholic	178 (40.4)	89 (42.6)	89 (38.5)	0.619	109 (38.2)	69 (44.5)	**0.052**
Protestant/Anglican	153 (34.8)	70 (33.5)	83 (35.9)		111 (38.9)	42 (27.1)	
Muslim	57 (13.0)	24 (11.5)	33 (14.3)		30 (10.5)	27 (17.4)	
Pentecostal/born again	43 (9.8)	23 (11.0)	20 (8.7)		28 (9.8)	15 (9.7)	
SDA	9 (2.0)	3 (1.4)	6 (2.6)		7 (2.5)	2 (1.3)	
Marital status							
Never married	9 (2.0)	2 (1.0)	7 (3.0)	**0.072**	9 (3.2)	0 (0.0)	**0.010**
Currently married/cohabiting	146 (33.2)	78 (37.3)	68 (29.4)		103 (36.1)	43 (27.7)	
Separated/divorced	85 (19.3)	44 (21.1)	41 (17.7)		46 (16.1)	39 (25.2)	
Widow/widower/single	200 (45.5)	85 (40.7)	115 (49.8)		127 (44.6)	73 (47.1)	
Current sexual partners							
None	285 (64.8)	134 (64.1)	151 (65.4)	0.310	183 (64.2)	102 (65.8)	0.883
One partner	131 (29.8)	60 (28.7)	71 (30.7)		87 (30.5)	44 (28.4)	
Two or more partner	24 (5.4)	15 (7.2)	9 (3.9)		15 (5.3)	9 (5.8)	
Livelihood activities							
Crop cultivation	254 (57.7)	97(46.4)	157(68.0)	**<0.001**	157 (55.1)	97 (62.6)	0.140
Business/petty trade	70 (15.9)	36(17.2)	34(14.7)		47 (16.5)	23 (14.8)	
Formal job/salarised	28 (6.4)	18(8.6)	10(4.3)		24 (8.4)	4 (2.6)	
Casual worker	50 (11.4)	34(16.3)	16(6.9)		31 (10.9)	19 (12.3)	
Others	38 (8.6)	24(11.5)	14(6.1)		26 (9.1)	12 (7.7)	

Table 2 results show that the majority, 71%, of participants engaged in intense vigorous activities such as brisk walking (>2 mph), shoveling, carrying heavy loads, and digging. At the same time, thirty-nine percent (39%) of older individuals living with HIV reported work that elevated their heart rates. Almost seventy percent (69%) of respondents were involved in activities like sweeping, clearing the compound, and gardening. Other reported activities included walking (34%), bicycling (17%), carrying farm produce (16%), and digging (59%). When asked about their weekly intake of fruits, meat, and vegetables, over half (55%) reported eating fruits for 1-3 days a week, 56% consumed vegetables 4 to 7 days a week, 56% ate red meat, and 54% ate white meat 1-3 days a week. A bivariate analysis using the chi-square test revealed significant associations between perceived quality of life and the following lifestyle factors: walking, cycling, carrying farm produce, travelling to and fro using a bicycle, weekly sports, recreation activities, eating white meat, and weekly white meat servings (all *p* <0.05).

**Table 2:** Quality of life and associated Lifestyle factors of older persons living with HIV

Perceived quality of living in older persons with HIV							
		Before			After		
Category	All	Normal	Poor	p-value	Normal	Poor	p-value
*Physical* *Activity*		N(%)	N(%)				
*Sweeping/raking/cleaning*	*305* *(69.3)*	*139* *(66.5)*	*166* *(71.9)*	*0.224*	*202* *(70.9)*	*103* *(66.5)*	*0.336*
*Walking*	*149* *(33.8)*	*94* *(45.0)*	*55* *(23.8)*	*<0.001*	*89* *(31.2)*	*60* *(38.7)*	*0.113*
*Cycling*	*26* *(5.9)*	*21* *(10.0)*	*5* *(2.2)*	*<0.001*	*20* *(7.0)*	*6* *(3.9)*	*0.181*
*Carrying* *farm* *produce*	*71* *(16.1)*	*46* *(22.0)*	*25* *(10.8)*	*0.001*	*53* *(18.6)*	*18* *(11.6)*	*0.057*
*Digging* *frequently*	*261* *(59.3)*	*123* *(58.9)*	*138* *(59.7)*	*0.850*	*166* *(58.2)*	*95* *(61.3)*	*0.535*
Daily work increases heart rate							
No	265 (60.2)	128 (61.2)	137 (59.3)	0.679	185 (64.9)	80 (51.6)	**0.006**
Yes	175 (39.8)	81 (38.8)	94 (40.7)		100 (35.1)	75 (48.4)	
Weekly vigorous activities							
None	125 (28.4)	58 (27.8)	67 (29.0)	0.201	90 (31.6)	35 (22.6)	0.125
1-3 Days	183 (41.6)	80 (38.3)	103 (44.6)		115 (40.4)	68 (43.9)	
4-7 Days	132 (30.0)	71 (34.0)	61 (26.4)		80 (28.1)	52 (33.5)	
In past 4 weeks, frequency							
3-4 times per week	229 (52.0)	110 (52.6)	119 (51.5)	0.742	151 (53.0)	78 (50.3)	0.770
1-2 times per week	89 (20.2)	44 (21.1)	45 (19.5)		59 (20.7)	30 (19.4)	
1-2 times per month	61 (13.9)	26 (12.4)	35 (15.2)		39 (13.7)	22 (14.2)	
Not at all	61(13.9)	29 (13.9)	32(13.9)		36 (12.6)	25 (16.1)	
Travel to & from places							
Using a bicycle/walking							
None	47 (10.8)	12 (5.7)	35 (15.2)	0.005	34 (11.9)	13 (8.4)	**0.022**
1-3 Days	156 (35.4)	75 (35.9)	81 (35.1)		88 (30.9)	68 (43.9)	
4-7 Days	237 (53.8)	122 (58.4)	115 (49.8)		163 (57.2)	74 (47.7)	
Recreation activities							
Sports, fitness & recreational							
No	379 (86.1)	169 (80.9)	210 (90.9)	**0.002**	249 (87.4)	130 (83.9)	0.311
Yes	61 (13.9)	40 (19.1)	21 (9.1)		36 (12.6)	25 (16.1)	
Weekly sports							
None	330 (75.0)	152 (72.7)	178 (77.1)	**0.003**	228 (80.0)	102 (65.8)	**<0.001**
1-3 Days	90 (20.5)	40 (19.1)	50 (21.6)		42 (14.7)	48 (31.0)	
4-7 Days	20 (4.5)	17 (8.1)	3 (1.3)		15 (5.3)	5 (3.2)	
Taking ARVS affects sports							
No	379 (86.1)	169 (80.9)	210 (90.9)	**0.002**	249 (87.4)	130 (83.9)	0.311
Yes	61 (13.9)	40 (19.1)	21 (9.1)		36 (12.6)	25 (16.1)	
Fruits consumption (Mangoes, passion fruits, guava)							
None	23 (5.2)	14 (6.7)	9 (3.9)	0.363	14 (4.9)	9 (5.8)	0.588
1-3 Days	242 (55.0)	118 (56.5)	124 (53.7)		153 (53.7)	89 (57.4)	
4-7 Days	134 (30.4)	61 (29.2)	73 (31.6)		93 (32.6)	41 (26.5)	
Not sure	41 (9.3)	16 (7.7)	25 (10.8)		25 (8.8)	16 (10.3)	
Vegetables consumption (Nakatti, Dodo, Buga, cabage)							
None	2 (0.4)	2(1.0)	0(0.0)	0.246	2 (0.7)	0 (0.0)	0.556
1-3 Days	158 (35.9)	79(37.8)	79(34.2)		99 (34.7)	59 (38.1)	
4-7 Days	245 (55.7)	115(55.0)	130(56.3)		163 (57.2)	82 (52.9)	
Not sure	35 (8.0)	13(6.2)	22(9.5)		21 (7.4)	14 (9.0)	
Red meat consumption (beef, mutton, goat meat)							
None	110 (25.0)	53(25.4)	57(24.7)	0.526	70 (24.6)	40 (25.8)	0.218
1-3 Days	245 (55.8)	113(54.1)	132(57.1)		158 (55.4)	87 (56.1)	
4-7 Days	17 (3.8)	11(5.3)	6(2.6)		15 (5.3)	2 (1.3)	
Not sure	68 (15.4)	32(15.3)	36(15.6)		42 (14.7)	26 (16.8)	
White meat (fish, chicken, duck)							
None	105 (23.8)	60(28.7)	45(19.5)	<0.001	70 (24.6)	35 (22.6)	0.196
1-3 Days	238 (54.1)	96(45.9)	142(61.5)		160 (56.1)	78 (50.3)	
4-7 Days	36 (8.2)	29(13.9)	7(3.0)		18 (6.3)	18 (11.6)	
Not sure	61 (13.9)	24(11.5)	37(16.0)		37(13.0)	24(15.5)	
Servings of white meat (weekly)							
*None*	164 (37.2)	86(41.1)	78(33.8)	<0.001	104(36.5)	60(38.7)	**0.028**
*Once*	169 (38.4)	60(28.7)	109(47.2)		122(42.8)	47(30.3)	
*Twice*	73 (16.6)	35(16.7)	38(16.5)		42(14.7)	31(20.0)	
*More* *than* *twice*	34 (7.7)	28(13.4)	6(2.6)		17(6.0)	17(11.0)	
Alcohol consumption							
No	*378* *(85.9)*	173 (82.8)	205 (88.7)	0.072	250 (87.7)	128 (82.6)	0.139
Yes	62 (14.1)	36 (17.2)	26 (11.3)		35 (12.7)	27 (17.4)	
Consumption rates (n=62)							
*Almost* *daily*	4 (6.4)	4 (11.1)	*0* *(0.0)*	*0.187*	*3* *(8.6)*	*1* *(3.7)*	*0.110*
*5-6* *days* *a* *week*	*3* (4.8)	3 (8.3)	*0* *(0.0)*		*3* *(8.6)*	*0* *(0.0)*	
*3-4* *days* *a* *week*	*5* (8.0)	3 (8.3)	*2* *(7.7)*		*5* *(14.3)*	*0* *(0.0)*	
*1-2* *days* *a* *week*	*12* (19.3)	5 (13.9)	*7* *(26.9)*		*7* *(20.0)*	*5* *(18.5)*	
*1-3* *days* *a* *month*	*19* (30.6)	12 (33.3)	*7* *(26.9)*		*9* *(25.7)*	*10* *(37.0)*	
*Less* *than* *once* *a* *month*	*19* (30.6)	9 (25.0)	*10* *(38.5)*		*8* *(22.9)*	*11(40.7)*	
Stopped alcohol due to ART							
*No*	*34* (54.8)	23 (63.9)	*11* *(42.3)*	*0.092*	*19* *(54.3)*	*15* *(55.6)*	*0.921*
*Yes*	*28* (45.2)	13 (36.1)	*15* *(57.7)*		*16* *(45.7)*	*12* *(44.4)*	
Smoking tobacco products							
No	*433* (98.4)	207 (99.0)	226 (97.8)	0.312	280 (98.2)	153 (98.7)	0.710
Yes	7 (1.6)	2 (1.0)	5 (2.2)		5 (1.8)	2 (1.3)	
High BP condition							
No	350 (79.5)	167 (79.9)	183 (79.2)	0.859	235 (82.5)	115 (74.2)	**0.040**
Yes	90 (20.4)	42 (20.1)	48 (20.8)		50 (17.5)	40 (25.8)	
Diabetes mellitus							
No	419 (95.2)	202 (96.7)	217 (93.9)	0.183	273 (95.8)	146 (94.2)	0.453
Yes	21 (4.8)	7 (3.3)	14 (6.1)		12 (4.2)	9 (5.8)	
Had heart disease							
No	430 (97.7)	205 (98.1)	225 (97.4)	0.631	279 (97.9)	151 (97.4)	0.749
Yes	10 (2.3)	4 (1.9)	6 (2.6)		6 (2.1)	4 (2.6)	

### Factors associated with perceived quality of life among older persons living with HIV

Table 3 presents the results of the multivariable analysis examining factors associated with poor perceived QoL among OPLHIV. Female older persons living with HIV had a 6% lower risk of poor QoL compared to males (aRR 0.94; 95% CI: 0.55 to 1.14). Those aged over 60 years were 1.1 times more likely to experience poor QoL compared to those aged 50-55 years (aRR 1.1; 95% CI: 0.65 to 1.71). The OPLHIV with secondary education had a 14% lower risk of poor QoL compared to those with no formal education (aRR 0.86; 95% CI: 0.16-0.58), while those with tertiary education had a 17% higher risk (aRR 1.17; 95% CI: 0.13-0.74). Married OPLHIV had a 35% lower risk of poor QoL compared to those who were unmarried (aRR 0.65; 95% CI: 0.42-1.01). Separated and widowed individuals had a 30% lower risk of poor QoL compared to those who were married (aRR 0.70; 95% CI: 0.45-1.46). Widowed or single individuals had a 15% lower risk of poor QoL compared to those who were married (aRR 0.85; 95% CI: 0.56-1.31). Unemployed OPLHIV had a 4% higher risk of poor QoL compared to those who were employed (aRR 1.04; 95% CI: 1.04-1.90). Individuals with one sexual partner had a 50% increased risk of poor QoL compared to those with no sexual partners (aRR 1.50; 95% CI: 1.16-1.93).

**Table 3:** Perceived quality of life by lifestyle factors among older persons living with HIV

	Before				After			
Variables	aRR	Lower	Upper	p-value	aRR.	Lower	Upper	p-value
*Gender*								
Male	**Ref.**				**Ref.**			
Female	.941	0.775	1.142	0.536	1.164	0.849	1.596	0.345
*Age*								
50–55 Years	**Ref.**				Ref.			
56–60 Years	1.178	0.946	1.471	0.142	1.076	0.786	1.474	0.648
>60 Years	1.178	0.947	1.464	**0.097**	1.343	0.982	1.837	**0.065**
*Education status*								
**None**	**Ref.**				**Ref.**			
Primary school	1.019	0.831	1.249	0.859	1.098	0.800	1.507	0.565
Secondary school	0.860	0.635	1.164	0.329	0.934	0.592	1.474	0.771
Tertiary/university	1.057	0.650	1.718	0.823	1.096	0.601	1.999	0.765
*Religious* *affiliation*								
**Roman catholic**	**Ref.**							
Protestant	1.101	0.915	1.355	0.339	0.881	0.635	1.221	0.446
Muslim	1.250	0.995	1.636	0.079	1.233	0.884	1.720	0.217
Pentecostal	0.956	0.699	1.305	0.774	1.040	0.673	1.607	0.860
SDA	1.891	1.112	3.214	**0.017**	0.387	0.106	1.414	0.151
*Employed*								
Yes	Ref.				**Ref.**			
No	1.409	1.041	1.906	0.026	0.928	0.677	1.270	0.639
*Livelihood* *activity*								
Crop cultivation	**Ref**				Ref.			
Business/Trade	0.861	0.619	1.199	0.377	0.842	0.553	1.259	0.402
Formal job/salarised	0.796	0.432	1.466	0.464	0.328	0.127	0.819	**0.017**
Casual worker	0.627	0.414	0.949	**0.027**	0.986	0.607	1.488	0.946
Others	0.637	0.424	0.955	**0.029**	0.783	0.474	1.248	0.304
*Marital status*								
Never married	**Ref.**							
Currently married	0.659	0.426	1.018	**0.060**	–	–	–	–
Separated/divorced	0.709	0.453	1.111	0.133	–	–	–	–
Widow/widower	0.856	0.560	1.310	0.474	–	–	–	–
*Sexual* *partners*								
None	**Ref.**				Ref.			
One	1.502	1.166	1.934	0.002	0.974	0.727	1.536	0.861
Two or three	1.072	0.631	1.821	0.796	1.110	0.602	2.241	0.738
*Smoking* *status*								
No	**Ref.**				Ref			
Yes	1.276	0.650	2.502	0.479	0.756	0.218	2.624	0.660
*Consume* *alcohol*								
No	**Ref.**				Ref			
Yes	0.713	0.509	0.983	**0.045**	1.272	0.900	1.798	0.173
*Weekly* *Physical* *E.*								
None	**Ref.**				Ref.			
1-3 days a week	1.002	0.805	1.247	0.865	1.119	0.774	1.618	0.549
4-7 days a week	0.832	0.653	1.060	0.136	1.384	0.967	1.983	**0.076**
*Weekly* *fruits*								
None	**Ref.**				Ref.			
1-3 days a week	1.272	0.791	2.043	0.321	0.796	0.434	1.461	0.590
4-7 days a week	1.447	0.893	2.346	0.134	0.709	0.363	1.384	0.183
Not sure	1.750	0.990	3.094	**0.054**	0.855	0.396	1.844	0.945
*Weekly* *Red* *Meat*								
None	**Ref.**				Ref.			
1-3 days a week	0.844	0.661	1.079	0.176	1.097	0.784	1.534	0.687
4-7 days a week	0.751	0.409	1.379	0.355	0.400	0.104	1.541	0.183
Not sure	0.735	0.492	1.098	0.133	0.981	0.575	1.675	0.945
*Weekly* *white* *meat*								
None	**Ref.**				**Ref.**			
1-3 days a week	1.615	1.239	2.106	<0.001	1.077	0.750	1.547	0.687
4-7 days a week	0.582	0.291	1.165	0.126	2.015	1.187	3.421	**0.009**
Not sure	1.340	0.905	1.985	0.144	1.194	0.711	2.005	0.503
*Weekly* *sports*								
None	Ref				Ref.			
1-3 days a week	1.156	0.910	1.468	0.234	1.944	1.479	2.556	**<0.01**
4-7 days a week	0.259	0.088	0.756	**0.013**	0.782	0.361	1.695	0.534
*Weekly* *walks*								
None	Ref.				Ref.			
1-3 days a week	0.843	0.651	1.091	0.195	1.261	0.747	2.129	0.385
4-7 days a week	0.791	0.620	1.008	**0.058**	1.003	0.599	1.695	0.992
*Diabetic*								
No	Ref				Ref.			
Yes	1.579	1.138	2.191	**0.006**	1.340	0.759	2.365	0.313
Constant	0.385	0.179	0.829	0.15	0.221	0.083	0.591	0.003

*** p<.01, ** p<.05, * p<.1

All bolded results are statistically significant

The results indicate that alcohol consumption was associated with a 29% reduced risk of poor quality of life (QoL) among OPLHIV compared to those who did not consume alcohol (aRR 0.71; 95% CI: 0.50-0.98). Inconsistent fruit consumption (1-3 days/week and 4-7 days/week) was associated with an increased risk of poor QoL (aRR 1.27; 95% CI: 0.79-2.04 and aRR 1.44; 95% CI: 0.89-2.34, respectively). Consuming red meat 1-3 days per week was associated with a 16% reduced risk of poor QoL (aRR 0.84; 95% CI: 0.66-1.07), while consuming red meat 4-7 days per week was associated with a 25% lower risk (aRR 0.75; 95% CI: 0.40-1.37). Consumption of white meat (such as fish, duck, rabbit, or chicken) 1-3 days per week was associated with a 61% increased risk of poor QoL compared to those who did not consume white meat (aRR 1.61; 95% CI: 1.23-2.10).

Engaging in walking for 4-7 days per week was associated with a 21% lower likelihood of poor QoL (aRR 0.79; 95% CI: 0.62-1.00). Additionally, older individuals engaging in sports 4-7 days per week had a 75% reduced risk of poor QoL compared to those who did not (aRR 0.25; 95% CI: 0.08-0.75). OPLHIV with diabetes had a 57% increased risk of poor QoL compared to those without diabetes (aRR 1.57; 95% CI: 1.13-2.19). No significant associations were found between poor QoL and level of education, religious affiliation, livelihood activities, vegetable consumption, other forms of weekly physical activity, or smoking.

## Discussion

This study aimed to assess perceived quality of life among older persons living with HIV (OPLHIV) in Uganda. The findings revealed a general improvement in quality of life among older individuals (≥50 years) following antiretroviral therapy (ART) initiation. This improvement likely results from ART’s ability to reduce viral load and enhance immune function, leading to physical health stability and subsequently, increased life satisfaction [22]. Furthermore, the initiation of treatment often fosters hope and a more positive outlook, potentially alleviating the psychological burden associated with HIV stigma and the fear of disease progression [12, 22]. However, this finding diverges from studies conducted in other regions, including Sub-Saharan Africa, Cambodia, South Africa, Nigeria, and Ethiopia [15, 24].

Our findings indicate no association between female gender and poor quality of life (QoL). This aligns with studies in Sub-Saharan Africa that suggest women experience better QoL outcomes [14, 15, 24]. This may be attributed to women’s proactive engagement with healthcare, as they tend to seek medical help earlier and demonstrate greater treatment adherence [14, 24]. Additionally, they often cultivate stronger social networks, which provide valuable emotional support. Furthermore, research suggests women may exhibit greater psychological resilience in the face of adversity [14]. Conversely, lower quality of life (QoL) among men may be influenced by job-related mobility and lifestyle choices, including alcohol consumption [14]. However, some studies also indicate that females experience better outcomes than males [24], suggesting a complex interplay of factors influencing QoL in both genders.

This study found that advanced age was associated with reduced quality of life, aligning with the established link between aging and heightened susceptibility to health problems and social isolation [2, 17]. Older adults living with HIV face a multitude of challenges, including age-related comorbidities and exacerbated social isolation, further complicated by multimorbidity, polypharmacy, frailty, and cognitive dysfunction [2]. These findings emphasize the need for age-specific interventions to improve mental health and QoL in older persons living with HIV [4, 17].

Although this study found no significant association between employment status and poor QoL among OPLHIV, existing research reveals a complex relationship. Employment is generally linked with improved physical and mental health related QoL, with a stronger effect on physical health [10]. Longitudinal studies suggest that employment can improve health outcomes, although healthier individuals are more likely to maintain employment [30]. Employed PLHIV report significantly higher overall QoL compared to their unemployed counterparts, especially those that have retired [7]. However, OPLHIV face substantial employment barriers, including potential loss of insurance, limited disability benefits, and discrimination due to frequent medical visits, can negatively impact QoL by hindering access to healthcare services, and breaching confidentiality [30]. Factors contributing to unemployment among PLHIV include older age, longer duration of HIV diagnosis, psychological distress and HIV-related illness [30]. Addressing perceived barriers and providing employment assistance could help improve overall QoL for PLHIV.

Our study found an association between marriage and poorer quality of life, a result that contrasts with some existing literature. Research on the relationship between marital status and quality of life among older persons living with HIV shows mixed results [4, 15, 21, 30]. Some studies suggest that being in a relationship, particularly marriage is linked to better QoL, highlighting the role of emotional support as a buffer against depression and anxiety in individuals with chronic health conditions, particulary those related to the stigma attached to HIV [4]. For instance, married older adults have reported higher QoL scores in psychological health and social relationships compared to divorced or widowed individuals [24]. Conversely, other research, including studies conducted in Nigeria, found associations between marriage and poorer QoL outcomes in this population [4, 7, 30]. This discrepancy, including the findings of our study, highlighst the complex and context-dependent nature of this relationship, suggesting that it may vary significantly across different regions and populations. Further research is needed to elucidate the underlying factors.

Our findings revealed a counterintuitive association: physically active older persons living with HIV (OLPHIV) reported poorer quality of life (QoL). This contrasts with existing literature demonstrating the positive impact of physical activity on QoL, mental well-being, and physical functioning in OPLHIV, particulary given their reduced aerobic capacity [20, 25, 30]. Studies consistently show that increased physical activity in this population leads to improvements in cardiorespiratory fitness, strength, and body composition [21, 30]. This discrepancy may stem from many Uganda HIV programs lack specific inclusion of OPLHIV, and tailored exercise programs that consider their unique physical capabilities and preferences are often absent. While reduced aerobic capacity occurs in PHIV, standard physical fitness assessments are safe for this population [6, 21]. Research indicates that PLHIA can achieve comparable health benefits from regular physical activity, regardless of their HIV status including improvements in cardiorespiratory fitness, oxygen pulse, maximum tidal volume, and a reduction in perceived stress [5, 21, 23, 30]. Therefore, the poorer QoL reported by physically active OPLHIV in our study likely reflects the challenges they face in accessing suitable exercise programs, rather than a negative impact of physical activity itself.

Our study found that weekly sport activities did not significantly influence poorer quality of life in older persons living with HIV. This does not negate the substantial evidence supporting the benefits of regular physical activity for this population. Research consistently demonstrates that moderate-intensity exercise, including aerobic and resistance training, can improve metabolic abnormalities, such as reducing excess fat, within as little as six weeks [6, 21]. Furthermore, it can alleviate ART side effectsby improving mood and reducing depressive symptoms [6, 21]. Exercise interventions have shown positive effects on CD+ cell count, HIV RNA viral load and HIV-specific biomarkers [5, 20, 25]. Regular physical activity is crucial for improving cardiorespiratory fitness, metabolic profiles, and overall quality of life in OLPHIV [21, 23, 29, 30]. Aerobic exercises performed three times weekly can effectively reduce HIV-associated depressive symptoms and anxiety by mitigating psychological disturbances [5, 11, 18, 21, 23]. While our study did not find a direct association between weekly sport activities and QoL it’s essential to emphasize that a wide range of physical activity, beyond those specifically assessed, can significantly contribute to the health and well-being of OPLHIV.

Our study found no association between vegetable or red meat consumption and poor QoL among older persons living with HIV (OLPHIV). However, PLHIV often experience metabolic disturbances, including high visceral fat mass and obesity influenced by poor dietary choices, sedentary lifestyles, chronic stress, and other lifestyle factors [2, 17]. OPLHIV frequently exhibit poor nutrition status, with many at risk of malnutrition and inadequate nutrient intake [2, 17]. A balanced diet is crucial for the overall health and well-being of OPLHIV [16]. Experts recommend a diet rich in fruits and vegetables to bolster the immune system [2], and adequate protein intake from sources like beans, meats, grains, and legumes to mitigate the long-term inflammatory effects of antiretroviral therapy (ART) on muscles [21]. lncorporating omega-3 fatty acids vitamin D, and B-12 can potentially enhance the effectiveness of antiretroviral therapy [1, 14, 21]. Inadequate food intake is linked to poorer QoL outcomes [15], while a healthy diet can reduce chronic disease risk and manage ART-related weight issues [4, 14, 16]. Food insecurity, particularly in rural areas, is associated with frailty in OPLHIV highlighting the need to address nutrition and physical activity in HIV management, aspects often inadequately addressed in routine care [21]. Therefore, implementing personalized nutritional plans and strategies for OPLHIV could significantly improve their health outcome and quality of life [19, 21].

Older persons living with HIV face unique health management challenges, particularly concerning ART drug interactions and nutrition [21]. ART can cause side effects like dyslipidemia, insulin resistance, and cardiovascular risks [30]. Polypharmacy, common due to increased lifespan and ART-associated comorbidities, further complicates management, potentially leading to nutritional and metabolic disorders like lipodystrophy, hyperglycemia, and bone changes [19, 27]. Nutritional interventions are crucial to mitigate ART side effects, such as diarrhea, nausea, and bone demineralization, and to improve ART adherence and effectiveness [21, 30]. Therefore, close monitoring for drug effects and toxicities, with consideration for non-HIV comorbidities, is essential when selecting ART regimens for this population [3]. In this study, common Ugandan staple foods were initially excluded due to widespread consumption. However, some older participants subsequently reported minor side effects when consuming these foods with ARVs [19], aligning with findings from other African ART studies.

Studies have shown that OPLHIV have lower physical activities compared to HIV-negative controls [5, 26]. Consequently, this irregular physical activity in OPLHIV may cause poor physical function and a diminished quality of life [26]. Despite the known benefits, many OPLHIV do not meet the required physical activity levels, exhibiting prolonged sedentary behavior [26]. However, research consistently demonstrate that aerobic exercise, resistance training, or a combination of both, is safe and effective in enhancing physical strength, improving body composition, and promoting psychological well-being in OPLHIV [26]. Moreover, physical activity and exercise interventions have been proven highly effective in improving cardiorespiratory fitness, metabolic profiles, and overall quality of life among people living with HIV [20, 25]. Notably, regular physical activity significantly reduces the risk of developing cardiovascular diseases, high blood pressure, type 2 diabetes, and several types of cancer, all of which can significantly impact individuals living with HIV [26]. Although research specifically focusing on OPLHIV is limited, evidence suggests that physical activity related interventions can significantly improve working capacity in this population [26]. In this study weekly physical activity 4 to 7 days a week was associated with poor QoL. So incorporating exercise into the care of OPLHIV is a safe and beneficial strategy for improving various health outcomes.

Early lifestyle modifications among OPLHIV, such as smoking cessation, weight management, alcohol avoidance, blood pressure control, and regular exercise, are crucial for achieving a longer lifespan and improved quality of life [16, 19,3 ]. A review of the HIV literature demonstrates that aerobic exercise intervention provides significant health benefits for people living with HIV [26]. Routine exercise, typically lasting at least 4-5 weeks with sessions of at least 40 minutes three times a week, has proved safe and effective [20, 25]. These benefits are seen across various exercise intensities, participant disease statuses, and current antiretroviral treatment regimens include improvements in cardiorespiratory fitness, body composition and psychological well-being by reducing adiposity, depression, and potentially increasing CD4 count [2, 11, 25].

### Strengths and limitations of the study

This study benefits from utilizing current data on dietary protein intake and physical exercise in older HIV-positive Ugandans and uses modified Poisson regression for risk ratios.

However, limitations include use of a cross-sectional design which gives a snapshot of QoL unlike longitudinal studies that would track changes over time providing a unique insight to OPLHIV. Unfortunately the QoL details on general health perception, physical functioning, role functioning, pain, health distress, mental health, social functioning, and energy/fatigue were captured but not included in this manuscript. Additional limitations are the reliance on potentially biased self-reported data, inability to establish causality, and potential regional variations due to multisite data collection.

### Conclusion and recommendations

ART initiation enhanced perceived QoL in OPLHIV.. We recommend integrating regional, home-based exercise and dietary consultations by physiotherapists throughout treatment to enhance well-being, address access barriers, and tailor interventions for sustained health improvemnents.

## Data Availability

Data is subject to third party restrictions.

## Abbreviations

OPLHIV: Older Persons living with HIV
ART: Antiretroviral therapy
AIDS: Acquired Immune Deficiency Syndrome
TASO: The AIDS Support Organization
MOH: Ministry of Health
UPHIA: Uganda Population Based HIV Impact Assessment
QoL: Quality of life
UNCST: Uganda National Council for Science and Technology
WHO: World Health Organization
